# Synthesis and Evaluation of Biologically Active Compounds from Heterocycles Class

**DOI:** 10.3390/molecules30020394

**Published:** 2025-01-18

**Authors:** Stefania-Felicia Barbuceanu, Octavian Tudorel Olaru

**Affiliations:** Faculty of Pharmacy, “Carol Davila” University of Medicine and Pharmacy, 6 Traian Vuia Street, 020956 Bucharest, Romania

## 1. Introduction

Heterocyclic compounds represent one of the most important classes of natural and synthetic compounds, playing essential roles in various fields, including medicinal chemistry. These compounds have attracted significant interest from researchers, particularly in the biomedical field, due to their immense therapeutic potential. In this broad class of compounds, five- and six-membered ring heterocycles containing one or more heteroatoms, such as nitrogen, oxygen, or sulfur, play a significant role in the metabolism of living cells [1,2,3,4].

Numerous derivatives of these heterocycles have demonstrated a wide spectrum of biological activities, including antibacterial, antifungal, anti-inflammatory, antioxidant, anticancer, antiviral, and antidiabetic properties [5,6,7,8,9]. Moreover, many of these compounds serve as fundamental scaffolds in the structure of bioactive molecules due to their ability to interact with specific biological targets, leading to important pharmacological effects [10]. Therefore, the majority of currently approved drugs contain one or more five- or six-membered heterocycles, either as isolated or fused rings. Statistical data reveal that over 85% of biologically active molecules are heterocycles or contain a heterocyclic ring, underscoring their critical role in drug design and development [11]. Moreover, recent research indicates that heterocycles are present in more than 90% of newly developed drugs [12].

Given the structural, physicochemical, and especially biological importance of heterocyclic compounds, this Special Issue intends to showcase new research in this field, with the aim to stimulate the development of bioactive molecules.

The Special Issue “Synthesis and Evaluation of Biologically Active Compounds from Heterocycles Class”, released between February 2022 and December 2023, presents 13 papers—9 research articles and 4 reviews—that make significant contributions to the field of heterocyclic compounds (according to the List of Contributions presented below).

## 2. An Overview of Published Articles

As an overview, the research results from the articles and reviews that have been published on this topic are briefly presented in the following paragraphs.

In the first article of this SI (contribution 1), Arshad et al. reported the synthesis and physicochemical properties of some phenoxyquinolines from the reaction of 6-bromoquinolin-4-ol and arylboronic acid derivatives, in the presence of Cu(OAc)_2_ or Cu(OAc)_2_·H_2_O, by Chan–Lam coupling, using different types of solvents (protic, aprotic, or mixed solvents) and bases (triethylamine, tetramethylethylenediamine or pyridine). The results indicated that the best yields were obtained when using the mixed solvent CH_3_OH/H_2_O (8:1) and triethylamine, in the presence of Cu(OAc)_2_·H_2_O as a catalyst. The antibacterial potential of synthesized compounds was investigated against ESBL-producing *E. coli* and methicillin-resistant *S. aureus*, and a molecular docking study was also performed, with the derivative containing 3,5-diclorophenyl moiety exhibiting the most promising potential.

The article by Wang et al. (contribution 2) focuses on evaluating the synthesis, characterization and antiproliferative activity against some cancer cells of a series of N14-aminoacid-tetrandrine derivatives optimized in order to improve the aqueous solubility and anticancer activity compared to tetrandrine (TE), a bis-benzylisoquinoline alkaloid known for its biological potential. This compound is used in medical therapy for treatment of silicosis, inflammatory pulmonary and cardiovascular diseases in China. Although its anticancer activity has recently been highlighted, further investigations are limited due to its poor aqueous solubility. Wang et al. synthesized new N14-amino acid-tetrandrine derivatives as potential antitumor agents through multi-step reactions starting from TE, and their structures were fully confirmed spectrally. The results of anticancer activity evaluations indicated that among all compounds, the 14-N-(L-proline)-tetrandrine displayed the best antiproliferative activity against human colorectal cancer (HCT-15) cells and the aqueous solubility was improved compared to TE. However, further studies are needed to confirm the antitumor potential of this compound.

In the article by Janowska et al. (contribution 3), the structural model chosen by the authors was the 1,2,4-triazole nucleus, knowing that it is present in the structure of various drugs currently in use, especially those with antifungal action. Also, such heterocycles are cited in the literature for their antifungal, antibacterial, antitumor, analgesic, anti-inflammatory, antiviral properties. On the other hand, Schiff bases are known for their antimicrobial, antiviral, cytostatic, anti-inflammatory, and antipyretic activities, the azomethine group being present in the structure of some drugs. Based on these findings, the authors synthesized, in a first stage, the 4-amino-5-(3-fluorophenyl)-2,4-dihydro-3*H*-1,2,4-triazole-3-thione intermediate by treatment of 3-fluorobenzoic acid hydrazide with carbon disulfide, in a potassium base medium, when the potassium salt of 3-fluorophenyldithiocarbazinic acid was obtained, followed by cyclization with hydrazine hydrate. Subsequently, by condensation with aromatic aldehydes, some new Schiff bases were synthesized, type of 5-(3-fluorophenyl)-4-[(arylidene)amino]-2,4-dihydro-3*H*-1,2,4-triazole-3-thione. The screening of the antibacterial and antifungal activity of the obtained compounds indicated that some derivatives exhibited a moderate antifungal activity against *C. albicans*, with higher activity exhibited by derivatives with a 4-methoxyphenyl moiety (MIC = 62.5 μg/mL). The cytotoxicity assays of the compounds against MDA-MB-231 and PC3 tumor cell lines indicated a low cytotoxic effect. To elucidate the mechanism of the antimicrobial activity of the tested compounds, molecular docking studies were conducted against topoisomerase IV, D-Alanyl-D-Alanine Ligase and dihydrofolate reductase.

In contribution 4, the research focused on the synthesis of new five- or six-membered heterocyclic compounds from the oxazolones or 1,2,4-triazinones class, compounds known for their wide biological potential as antifungal, antibacterial, antitumor, anti-inflammatory compounds, etc., by grafting onto their molecule a diphenylsulfone moiety, also with antibacterial, antitumor, antioxidant, and antiviral properties. The synthesis of oxazol-5(4*H*)-ones involved the condensation of 2-(4-(4-X-phenylsulfonyl)benzamido)acetic acid intermediates with benzaldehyde or 4-fluorobenzaldehyde, in acetic anhydride and in the presence of sodium acetate. Subsequently, the reaction of oxazolones with phenylhydrazine, in acetic acid and sodium acetate, yielded corresponding 1,2,4-triazin-6(5*H*)-ones. The toxicity of new compounds, whose structure was confirmed by current spectral techniques, was tested on *Daphnia magna* Straus crustaceans and on the budding yeast *Saccharomyces cerevisiae*. The screening results against *D. magna* indicated that the toxicity depends on both the type of heterocycle and the nature of the halogen, oxazolones being less toxic than triazinones and those against *S. cerevisiae* indicated that the action of the compounds is considerably hindered by the activity of the MDR transporters Pdr5 and Snq2. The predictive studies indicated that the newly synthesized derivatives could inhibit cancer cell proliferation by targeting certain protein kinases, particularly PDGF-R and FAK2.

The article by Sharma et al. (contribution 5) presents the synthesis of a new series of nitrostyrene-based spirooxindoles by the reaction of isatin or 5-bromoisatin with different amino acids and (E)-2-aryl-1-nitroethenes derivatives in a chemo/regio-selective manner through a [3+2] cycloaddition (Huisgen) reaction under microwave irradiation, with the structure elucidated by ^1^H- and ^13^C NMR, HRMS and by spectral analysis single crystal X-ray crystallographic study in case of one compound. The 23 spiro[pyrrolidine-2,3′-oxindoles] derivatives were screened for in vitro anticancer activity against human lung (A549) and liver (HepG2) cancer cell lines with promising results. Some of these compounds were found to be more efficient and selective than standard references such as artemisinin and chloroquine.

In the next article (contribution 6), Yu et al. investigated the effects of aseptic inflammation and heavy metal exposure on immune responses, as well as the potential immunomodulatory properties of a new 1-[1-(2,5-dimethoxyphenyl)-4-(naphthalene-1-yloxy)but-2-yn-1-yl]-4-methylpiperazine complexed with cyclodextrin. The new 4-naphthoxy-butynylamine derivative was obtained through the aminomethylation reaction between 1-(prop-2-ynyloxy)naphthalene with 2,5-dimethoxybenzaldehyde and 1-methylpiperazine in dioxane and in the presence of copper(I) iodide. The results of the study suggest that the complex may possess immunomodulatory properties, especially in the context of aseptic inflammation and heavy metal exposure.

In the next article (contribution 7), Tian et al. addressed the need to develop more effective antifungal agents for the eradication of fungal diseases of crops that pose a serious threat to their production and quality globally. Taking into account the excellent antifungal properties of compounds with the thiazole structure, the authors proposed to obtain new compounds from this class that contain in their molecule a new pharmacophore center, the hydrazone fragment, also known for its antifungal activity. Thus, 42 new thiazole derivatives containing an acylhydrazone moiety were obtained by the condensation of 2-aryl-thiazol-4-yl carboxylic acid hydrazide with different aromatic aldehydes, in ethanolic medium and acetic acid, under reflux. The structures of these compounds were confirmed using spectral techniques. The antifungal activity of the synthesized compounds was screened against *Magnaporthe oryzae*, *Colletotrichum camelliaet*, *Bipolaris maydis*, and *Sclerotinia sclerotiorum*, and the results revealed that most of these derivatives exhibited antifungal activities against *M. oryzae* and *C. camelliaet* at 25 μg/mL. Some of them had a superior effect to the commercial fungicides (isoprothiolane or phenazine-1-carboxylic acid) against *M. oryzae.* Regarding the structure–activity relationship, the results suggested that the presence of some halogens or methyl/methoxy groups grafted on the phenyl moieties in ortho or para position can improve the antifungal activity against *M. oryzae*.

Research carried out by Menozzi et al. (contribution 8) was directed towards the synthesis and evaluation of the activity against *Trypanosoma cruzi* of some triazole and imidazole derivatives as drug candidates for Chagas disease (CD) treatment, knowing that it remains the leading cause of death from parasitic diseases in America. The synthesis involved a Friedel–Craft acylation reaction of 1,3-difluorbenzene with chloroacetyl chloride, in the presence of aluminum chloride, obtaining 2-chloro-1-(2,4-difluorophenyl)ethanone. This intermediate, when treated with an azole type of 3-nitro-1*H*-1,2,4-triazole, 2-nitroimidazole or 1,2,4-triazole, through a nucleophilic substitution reaction, led to carbonyl derivatives. The reduction of the last azole derivatives with sodium borohydride generated hydroxylated azole derivatives. The screening of the obtained compounds against *T. cruzi* indicated that 1-(2,4-difluorophenyl)-2-(3-nitro-1*H*-1,2,4-triazol-1-yl)ethanol derivative was 4-fold more potent and 2-fold more selective compared to benznidazole (BZN), the reference drug. This derivative was not mutagenic at the concentrations tested, showed a favorable in silico ADMET profile, and a low hepatotoxicity according to the high values of CC50 in HepG2 cells. In addition, the same derivative showed a higher rate of conversion by nitroreductase, being metabolized three times faster compared to BZN, at a concentration of 50 μM.

In the next paper (contribution 9), Carreiro et al. focused on identifying new anti-Alzheimer agents, taking into account the urgent need for effective treatments for this disease, which continues to affect a growing number of patients. Considering that both quercetin and 1,2,3-triazole derivatives have anti-Alzheimer properties, a library of 21 quercetin-1,2,3-triazole hybrids was screened for their cholinesterase (AChE and BuChE) inhibitory activity, the results indicating that most of them were effective for eqBuChE inhibition. The determination of the IC_50_ values indicated that some of them were more potent than galantamine, a derivative with isatin unit and methoxy groups that is more potent even than quercetin. In order to understand the action mechanism of derivatives that showed very good to excellent inhibitory activity against eqBuChE, kinetic studies were performed. The investigation of these quercetin-1,2,3-triazole hybrids’ effect on H_2_O_2_-induced oxidative damage in cultured MCF-7 cells indicated the same isatin derivative, and also two others having cyclopropyl/2-hydroxypropan-2-yl moiety and free-hydroxy groups in the molecule did not affect viability (at 12.5 μM) and displayed a protective effect against oxidative stress induced by hydrogen peroxide in cell damage in MCF-7 cells. Moreover, the toxicity evaluation for the most promising derivatives on *Artemia salina* indicated a low toxicity.

The review by Angeli et al. (contribution 10) presents a detailed analysis of the literature data over the last years on the progress of the five-membered heterocyclic sulfonamides with O and/or N either with S or S and N referring to their synthesis and biological activity, as a useful scaffold for obtaining new carbonic anhydrase inhibitors. The choice of five-membered heterocyclic sulfonamides as a field of research was determined by their superior biological activity, experimentally proven, compared to those with a six-membered heterocyclic nucleus. Thus, research and results of various sulfonamides containing different heterocyclic rings such as benzofuran, indole, pyrrole, furazan, furoxane, isoxazole, thiophene, thiazole, benzothiazole, thiadiazole were presented. The review emphasizes five-membered heterocycle sulfonamides with S and N heteroatoms from the 1,3,4-thiadiazole class with important pharmacological properties as agents in anticancer therapy, as anti-infective agents, in CNS diseases, or as anti-obesity experimental agents. Furthermore, 1,3,4-thiadiazole sulfonamides are particularly significant as some derivatives are already used in medical therapy as clinically approved drugs, such as acetazolamide and methazolamide among other derivatives.

The study by Nitulescu et al. (contribution 11) reviewed the use of small molecules as protein kinases inhibitors (PKIs), a major focus in oncology. The pyrazole ring is extensively utilized in medicinal chemistry and drug development. This scaffold is considered a privileged structure due to its synthetic accessibility, drug-like properties, and versatile bioisosteric replacement function. It also plays a key role in many PKIs, including inhibitors of Akt, Aurora kinases, MAPK, B-raf, JAK, Bcr-Abl, c-Met, PDGFR, FGFR, and RET. The review focused on the importance of the unfused pyrazole ring within clinically tested PKIs and the additional required elements of the chemical structures. The authors identified a number of 42 compounds that were clinically tested PKIs and contained an unfused pyrazole ring, highlighting its importance compared to other scaffolds such as the imidazole (10 compounds) and pyrrole rings (7 compounds). The pyrazole scaffold offers several advantages in PKI design, including its aromatic nature, ability to act as a hydrogen bond acceptor or donor, and its participation in π-stacking interactions with aromatic residues in kinase active sites, which enhances binding affinity. These compounds inhibit a diverse range of PKs, with many showing relative selectivity toward specific or closely related kinases, underscoring the usefulness of the pyrazole ring in PKI development. The structure and mechanism of action were also discussed, indicating that the pyrazole ring can function as an ATP analog, competitively binding to its site, or as a linker to facilitate the proper conformation for inhibition. However, the authors conclude that it is crucial to consider the overall structure and composition of these inhibitors, as additional structural elements can significantly contribute to their activity and selectivity.

The review by Leonte et al. (contribution 12) presents the synthesis and biological properties of flavones and their related flavonoid compound type of flavonols and aurones. The focus on these classes of compounds is driven by their numerous biological properties, including antioxidant, anti-inflammatory, antibacterial, antiviral and anticancer properties. The paper presents extensive literature data on the synthesis methods of flavones and aurones by derivatization and modulation of the structure in order to enhance the biological properties for development of new effective anticancer and antimicrobial agents. The most applied methods for the synthesis of flavones, hydroxyflavones and aurones involve the cyclization of o-hydroxychalcones, the products obtained depending not only on the reaction conditions and the catalysts used, but also on the nature and position of the substituents on the aromatic rings. The review also discusses the probable mechanisms involved in the synthesis of these compounds. Also, in this review, various synthetic analogs of flavones or aurones with antitumor, antibacterial, antifungal and antiviral activity are highlighted.

The paper by Buchanan et al. (contribution 13) reviewed the recent progress obtained in click chemistry that uses a simple and efficient method to join two molecular building blocks under mild and adaptable conditions, resulting in a new structure in high yields, requiring minimal purification. The paper discusses the literature data related to the hybridization of complex structures, alkaloids, with a five-membered heterocycle, 1,2,3-triazole, in order to obtain new structures with superior biological activity. This 1,2,3-triazole scaffold is a known pharmacophore, present in the molecule of various FDA-approved drugs such as carboxyamidotriazoles (calcium channel blocker), rufinamide (anticonvulsant), cefatrizine, tazobactam, and radezolid (antibiotics). The authors employed computational methods to evaluate the ADMET properties and drug-like qualities of hybrid molecules like alkaloid-1,2,3-triazole.

## 3. Conclusions

This Special Issue presents a collection of articles dedicated to research in the field of five- and six-membered heterocyclic compounds. The studies focus on the synthesis and/or evaluation of the biological properties of various five-membered heterocyclic compounds, including 1,2,4-triazoles, oxazoles, thiazoles, 1,2,3-triazoles, imidazole or spirocyclic structures such as spiro[pyrrolidine-2,3′-oxindoles] as well as compounds featuring six-membered heterocyclic systems like quinolines, isoquinolines, triazines, and piperazines.

These investigations have enriched the field, making important contributions to the understanding and development of these compounds.

The review articles in this Issue highlight significant advances and current topics in research areas, including the following: 1. five-membered heterocyclic sulfonamides as scaffolds for the synthesis of new carbonic anhydrase inhibitors, with a focus on 1,3,4-thiadiazole derivatives and their pharmacological/clinical applications; 2. the role of pyrazole-containing molecules as protein kinase inhibitors; 3. the synthesis and biological activities of flavones, along with related flavonoid compounds such as flavonols and aurones; and 4. molecular hybridization of alkaloids incorporating 1,2,3-triazole cores via click chemistry.

All these contributions offer valuable insights and guidance for researchers interested in heterocyclic chemistry. However, the increasing demand for new heterocyclic molecules with improved bioactivity compared to existing compounds shows the need to continue and expand research on their synthesis, physicochemical properties, and their biological evaluation in order to identify novel therapeutic agents.

This Special Issue underscores the decisive role of heterocyclic compounds in advancing medicinal and synthetic chemistry. The studies presented here not only highlight the diversity and potential of these molecular frameworks, but also demonstrate the interdisciplinary efforts needed to uncover their applications. As the challenges of drug resistance and unmet therapeutic needs continue to increase, the findings presented in this Issue represent the basis of future innovations and breakthroughs in heterocyclic chemistry.
